# Hominoid-specific retrotransposons fuel regulatory novelty in early brain development

**DOI:** 10.1016/j.xgen.2025.101038

**Published:** 2025-10-08

**Authors:** Larisa Okorokova, Gael Cristofari

**Affiliations:** 1 Université Côte d’Azur, Inserm, CNRS, Institute for Research on Cancer and Aging of Nice (IRCAN), Nice, France

## Abstract

Transposable elements can drive genetic innovation. In this issue of *Cell Genomics*, Adami et al.^1^ investigate the impact of hominoid-specific L1 retrotransposons on early embryonic and brain development. Using induced pluripotent stem cells and cerebral organoid models, multi-omics, and CRISPRi-mediated silencing, they uncover a *cis*-regulatory role for these young retrotransposons in early human brain development.

## Teaser

Transposable elements can drive genetic innovation. In this issue of *Cell Genomics*, Adami et al. investigate the impact of hominoid-specific L1 retrotransposons on early embryonic and brain development. Using induced pluripotent stem cells and cerebral organoid models, multi-omics, and CRISPRi-mediated silencing, they uncover a *cis*-regulatory role for these young retrotransposons in early human brain development.

## Main text

Transposable elements (TEs) occupy substantial portions of eukaryotic genomes. While their mobility can threaten genomic integrity, the accumulation of mutations and millions of years of coevolution with their host genomes have often neutralized them. Some have even been co-opted for critical physiological functions in a form of genomic upcycling. This evolutionary process has led to remarkable innovations; for instance, TE-derived proteins gave rise to the recombination-activating gene (RAG) enzymes that built the adaptive immune system in vertebrates and the syncytins essential for placenta formation. Beyond providing new protein-coding genes, TEs can also be co-opted as regulatory RNAs or *cis*-regulatory elements, supplying new promoters and enhancers and even shaping the 3D structure of the genome.^2^ Particularly fascinating is the convergent nature of some of these events, with independent insertions from distinct TE families across different evolutionary lineages that ultimately contribute to the same function. However, most documented cases, particularly in vertebrates, involve ancient TE families that expanded deep in evolutionary time. The work by Adami et al. in this issue of *Cell Genomics* now investigates whether much younger TE families, such as hominoid-specific LINE-1 (L1) retrotransposons (L1HS, L1PA2, L1PA3, and L1PA4), could have a functional impact on human pluripotent stem cells and neural development.^1^

L1 retrotransposons are the only autonomously active TE family in humans, accounting for 17% of our genome. While their potential to shape gene regulatory networks is well established, this activity is tightly regulated.^2^ For instance, during a wave of DNA demethylation in early development, both L1 transcription and L1-associated *cis*-regulatory elements are transiently activated, before being re-silenced in most somatic tissues. The brain, however, is a striking exception, where L1s remain expressed and can even retrotranspose, generating somatic mosaicism.^3^^,^^4^ Despite this knowledge, the collective impact and functional importance of young, hominoid-specific L1s in human early embryonic and brain development have remained unclear. This question is particularly challenging to address with conventional animal models. Although rodents and other mammals have their own active L1s, the regulatory promoter regions of young, hominoid-specific L1s have diverged substantially. Crucially, the recent expansion of these families in the hominoid lineage has scattered thousands of unique insertions across the genome, creating a landscape of potential *cis*-regulatory elements that is absent in other species.

To address this challenge, Adami et al. turned to cerebral organoids derived from human induced pluripotent stem cells (hiPSCs) as a 3D model for early human brain development. Their multi-omics approach is particularly noteworthy, combining short- and long-read RNA sequencing with advanced epigenomic profiling. Adopting an integrated strategy recently pioneered to resolve L1 activity at high resolution,^5^ they could accurately profile the transcriptional activity of individual L1 promoters rather than just measuring aggregate global L1 expression. This detailed view confirmed young L1 expression in hiPSCs^6^ and neuronal progenitor cells.^3^ More profoundly, it revealed that hominoid-specific L1s exert *cis*-acting effects by functioning as alternative promoters for almost a hundred genes in hiPSCs. Many of these L1-regulated genes, such as *ELAPOR2*, *PPP1R1C*, and *PLCB1*, are implicated in brain development. L1-driven transcripts are also present in developing human fetal forebrain tissue, suggesting a functional role *in vivo*. Such co-option of L1 promoters appears to be a recurring evolutionary strategy, as similar chimeric transcripts were recently found to play a functional role in shaping the morphological complexity of mouse parvalbumin interneurons.^7^

**Figure 1:**
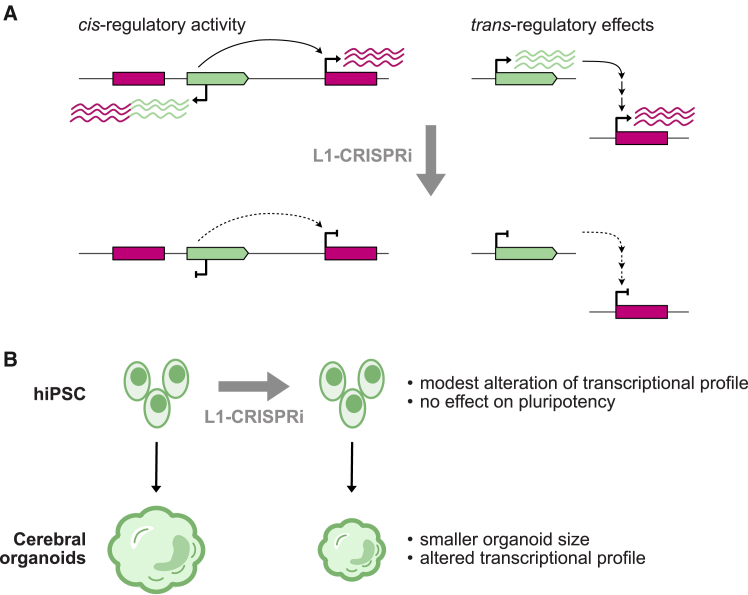
Regulatory and developmental consequences of L1 silencing (A) CRISPRi silencing of hominoid-specific L1s leads to complete suppression of their potential *cis*- and *trans*-regulatory functions. (B) Consequences of L1 silencing on pluripotency and early human neurodevelopment *in vitro* models.

Moving beyond an in-depth characterization, Adami et al. have directly tested the functional importance of young L1 expression. They achieved this by silencing L1s *en masse* in hiPSCs and human cerebral organoids using an optimized CRISPR interference (CRISPRi) system that specifically targets the internal promoter of young, full-length L1s (Figure 1A). This strategy was particularly thorough. First, RNA sequencing (RNA-seq) and mass spectrometry proteomics confirmed efficient and specific knockdown. Second, the method suppressed both sense and antisense promoter activity of L1s, which is critical, as the latter frequently drives chimeric transcript expression. Finally, because L1s can function as enhancers,^2^ this CRISPRi approach implicitly blocked their potential long-range regulatory activity as well.

Adami et al. first applied their CRISPRi system to hiPSCs. Despite achieving virtually complete L1 silencing, they detected no effect on pluripotency: the cells still expressed pluripotency markers and could differentiate into all three germ lineages (Figure 1B). These findings may seem to contradict previous reports suggesting that L1s are essential for early development.^2^ However, those earlier studies focused on the earliest stages of embryogenesis, specifically the exit from the totipotent naive state (e.g., post-zygotic genome activation at the 8-cell stage in humans). In contrast, the hiPSCs used in this study were already in a primed pluripotent state. Thus, the different outcomes likely reflect the distinct developmental windows being examined.

The impact of L1 silencing became clear when the hiPSCs were differentiated into cerebral organoids (Figure 1B). Compared to controls, these L1-deficient organoids showed a modest but significant reduction in size, without any change in their overall cellular composition. This phenotype was associated with transcriptional changes within neuronal progenitor cells (NPCs), which form the most abundant cell population in the organoids. Notably, L1 silencing led to the upregulation of numerous genes linked to neuronal differentiation and neurogenesis. The authors therefore conclude that young L1s help maintain the multipotent state of NPCs, while silencing them promotes premature differentiation, which, in turn, impairs overall organoid growth. This conclusion is consistent with previous works employing short hairpin RNA (shRNA)-mediated L1 suppression in human cortical organoids and in the developing mouse brain,^8^^,^^9^ which found that L1 expression can also regulate neural migration and maturation at later developmental stages. Furthermore, while this organoid model primarily contains NPCs, L1s have also been implicated in gliogenesis in the mouse brain,^8^ suggesting that they may regulate multiple cell lineages during brain development.

Dissecting L1 function with loss-of-function experiments is not straightforward, as the choice of tool determines which molecular mechanisms are interrogated. The CRISPRi approach used by Adami et al. is comprehensive, repressing both *cis*-acting mechanisms (promoter and enhancer activity) and potential *trans*-acting mechanisms mediated by L1-derived RNA species. This approach also silences chimeric transcripts driven by the L1 antisense promoter. Alternative tools offer more targeted perturbation: shRNAs primarily degrade cytoplasmic L1 mRNA (though some nuclear effects have been reported),^9^ antisense oligonucleotides degrade nuclear L1 RNAs (*trans*) without affecting L1-derived *cis*-regulatory elements, while reverse transcriptase inhibitors block retrotransposition itself. A challenge for all sequence-based tools is the heterogeneity of L1 copies, though the low divergence of young families makes them easier to target uniformly. Thus, combining multiple approaches is essential for a complete understanding of retrotransposon function.^10^

While a role for young L1s in early neurogenesis is established in this study, their impact on the later, complex processes of brain maturation remains an open question. Probing this would require evolving the CRISPRi system to make it inducible and applying it to organoids cultured for longer periods, which develop a richer diversity of cell types, including astrocytes and microglia. Future studies in these engineered “mini brains” could explore how L1 silencing affects neuronal connectivity, electrophysiological activity, and detailed cell morphology, providing a deeper understanding of how these young retrotransposons have shaped the hominoid brain.

Accumulating evidence from diverse experimental models points to a critical role for L1s in cerebral development, though the precise molecular mechanisms remain unclear and likely cooperate. One model posits that L1 RNA acts in *trans* as a non-coding RNA sequestering the Polycomb complex and preventing H3K27me3 deposition and heterochromatin formation on genes relevant to neuronal differentiation and maturation.^9^ In contrast, the findings by Adami et al. support a model where L1 insertions function as alternative promoters or enhancers for neurodevelopmental genes. This raises a key question: what common feature drives the convergent phenotypes of L1 suppression in mouse and human models, despite the lineage-specific nature and expansion of young L1 elements? Perhaps the answer lies in the fact that these are simply the most transcriptionally active L1 families in the neuronal progenitors of their respective species.

The *cis*-acting model raises an additional question: is the organoid phenotype driven by the cumulative contribution of many L1s or by a few critical loci? This question is particularly intriguing given previous work from Jakobsson’s group, which showed that silencing a single human-specific L1-derived long non-coding RNA (*LINC01876*) produces a similar reduction in organoid size.^4^ Individual variation also deserves further investigation, as polymorphic L1s may constitute an underappreciated source of neurodevelopmental diversity and disorders, requiring studies with greater genetic diversity than the two current hiPSC lines.

Proper brain development may depend on a precise balance of L1 expression. The misexpression of neurogenesis-linked genes in L1-silenced organoids suggests that too little L1 activity could lead to neurological defects. Conversely, too much L1 expression may be equally detrimental. Rett syndrome, for instance, is a neurological disorder linked to mutations in *MeCP2*, an L1 repressor, and is characterized by increased neuronal L1 activity.^2^ Future gain-of-function studies, perhaps using CRISPRa or MeCP2 knockout organoids, will provide crucial insights into how recently evolved retrotransposons contribute to human-specific aspects of brain development and disease.

## Acknowledgments

This work was supported by the Agence Nationale de la Recherche (Idex UCAJEDI, ANR-15-IDEX-01; ActiveLINE, ANR-21-CE12-0001), Region Sud and Inserm (MobileGenomAI), Inserm (GOLD), and CNRS (GDR 3546).

## Declaration of interests

G.C. is an unpaid associate editor of the journal *Mobile DNA* (Springer Nature) and, in the last 5 years, has received honoraria from EssilorLuxottica for an educational event and from Ono Pharma for consultancy.

## Declaration of generative AI and AI-assisted technologies in the writing process

During the preparation of this manuscript, the authors used a combination of Le Chat (Mistral), Claude Sonnet 4 (Anthropic), Gemini 2.5 Pro (Google), and Chat GPT-5 (OpenAI) for grammatical error correction and stylistic changes. The authors thoroughly reviewed and edited the content following the use of these tools and take full responsibility for the final content of the publication.
